# Post-Messinian evolutionary relationships across the Sicilian channel: Mitochondrial and nuclear markers link a new green toad from Sicily to African relatives

**DOI:** 10.1186/1471-2148-8-56

**Published:** 2008-02-23

**Authors:** Matthias Stöck, Alessandra Sicilia, Natalia M Belfiore, David Buckley, Sabrina Lo Brutto, Mario Lo Valvo, Marco Arculeo

**Affiliations:** 1 University of California, Berkeley, Museum of Vertebrate Zoology, 3101 Valley Life Sciences Building #3160, Berkeley, CA 94720-3160, USA; 2 University of Palermo, Dipartimento di Biologia Animale, Via Archirafi, 18, 90123 Palermo, Italy; 3 Dept. Ecology and Evolution, University of Lausanne, Biophore, CH-1015 Lausanne, Switzerland

## Abstract

**Background:**

Little attention has been paid to the consequences of the last landbridge between Africa and Sicily on Mediterranean biogeography. Previous paleontological and scarce molecular data suggest possible faunal exchange later than the well-documented landbridge in the Messinian (5.3 My); however, a possible African origin of recent terrestrial Sicilian fauna has not been thoroughly tested with molecular methods. To gain insight into the phylogeography of the region, we examine two mitochondrial and two nuclear markers (one is a newly adapted intron marker) in green toads (*Bufo viridis *subgroup) across that sea barrier, the Strait of Sicily.

**Results:**

Extensive sampling throughout the western Mediterranean and North Africa revealed a deep sister relationship between Sicilian (*Bufo siculus *n.sp.) and African green toads (*B. boulengeri*) on the mitochondrial and nuclear level. Divergence times estimated under a Bayesian-coalescence framework (mtDNA control region and 16S rRNA) range from the Middle Pliocene (3.6 My) to Pleistocene (0.16 My) with an average (1.83 to 2.0 My) around the Pliocene/Pleistocene boundary, suggesting possible land connections younger than the Messinian (5.3 My). We describe green toads from Sicily and some surrounding islands as a new endemic species (*Bufo siculus*). *Bufo balearicus *occurs on some western Mediterranean islands (Corsica, Sardinia, Mallorca, and Menorca) and the Apennine Peninsula, and is well differentiated on the mitochondrial and nuclear level from *B. siculus *as well as from *B. viridis *(Laurenti), whose haplotype group reaches northeastern Italy, north of the Po River. Detection of Calabrian *B. balearicus *haplotypes in northeastern Sicily suggests recent invasion. Our data agree with paleogeographic and fossil data, which suggest long Plio-Pleistocene isolation of Sicily and episodic Pleistocene faunal exchange across the Strait of Messina. It remains unknown whether both species (*B. balearicus, B. siculus*) occur in sympatry in northern Sicily.

**Conclusion:**

Our findings on green toads give the first combined mitochondrial and nuclear sequence evidence for a phylogeographic connection across the Strait of Sicily in terrestrial vertebrates. These relationships may have implications for comparative phylogeographic research on other terrestrial animals co-occurring in North Africa and Sicily.

## Background

### On the phylogeographic patterns of terrestrial Sicilian fauna

**Figure 1:**
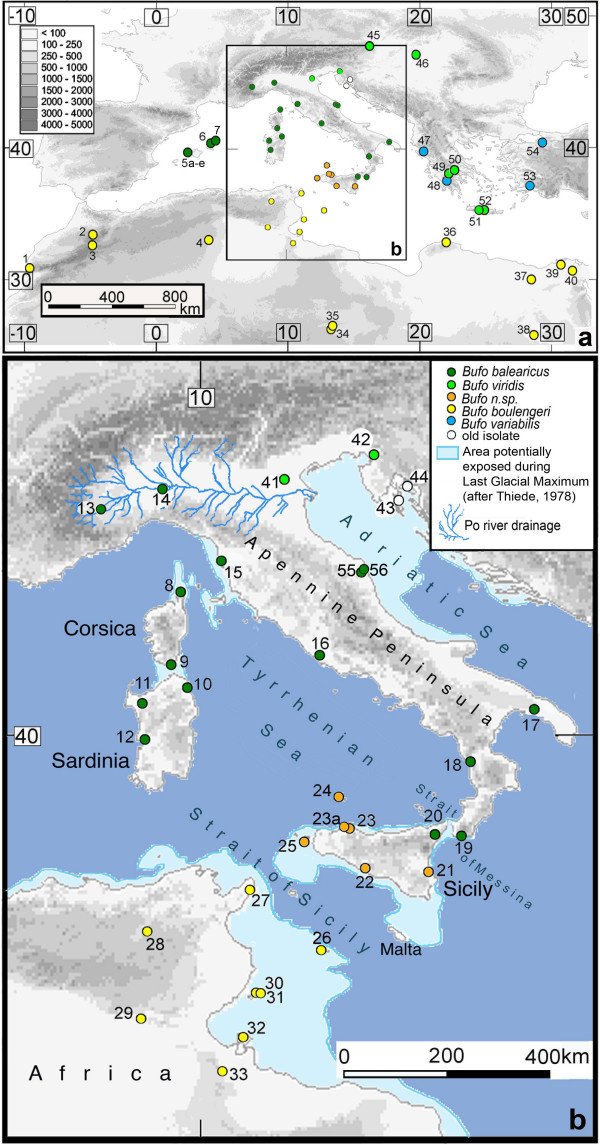
**Study area in the central and western Mediterranean**. (a) Mediterranean with sampling localities as in Additional file 1, (b) Enlarged rectangle (b) from (a) with approximated paleo-coastline during the Last Glacial Maximum [after 7].

Since Busack [1,2], the faunal relationships between North Africa and Iberia have been the focus of numerous molecular phylogeographic studies, yet little attention has been drawn to the consequences that the last landbridge between Africa and Sicily may have had on Mediterranean biogeography. As was the Strait of Gibraltar, the Strait of Sicily (no universally accepted name in English; other names include Sicilian Strait, Sicilian Channel, Channel of Sicily, Pantelleria Channel) is purported to have formed at the end of the Messinian salinity crisis (5.3 Mya), at the Miocene/Pliocene boundary [e.g. [3-6], F. Rögl pers. comm.]. The Messinian [4] was a geological period from 5.59 to 5.33 Mya during which the Mediterranean Sea was isolated from the Atlantic Ocean, resulting in a large decrease in the Mediterranean Sea level and the formation of landbridges between Africa, Europe and most Mediterranean islands. This included a well-documented landbridge between Africa and a landmass that later became part of Sicily, which may be the last terrestrial connection between the African mainland and the island. However, while it is known that Sicily and Tunisia are at present approximately 140 km apart, low Pleistocene sea levels of about -120 m [7-9] would have repeatedly drawn the north African paleo-coast and the Sicilian landmass closer than ~50 km (Figure 1). In addition, current shoals [10,11] may be remnants of Pleistocene "stepping stone islands" that may have facilitated terrestrial animals, including humans [12], in overcoming the sea barrier. Therefore, the phylogenetic depths of sister relationships between Sicily and Africa may vary as they do for trans-Gibraltarian relationships [2,13]. Observations of this pattern in widespread taxa might be explained by multiple invasions across the Strait of Sicily and/or additional potential invasions from the region that now forms the Italian (Apennine) Peninsula.

As expected for an island with a long Pliocene isolation [14], endemic (island specific) forms in several animal groups on Sicily have been revealed by molecular analyses (e.g.: hedgehogs, *Erinaceus *[15]; shrews,* Crocidura *[16]; mice, *Apodemus *[17]; beetles, Pachydemidae [18]; terrapins,* Emys *[19]; skinks, *Chalcides *[20]). Using these methods, some of these species have been shown to be sister taxa to populations from the Apennine Peninsula (e.g. *Apodemus, Erinaceus*) or to be of very recent Apennine peninsular (or Calabrian) origin (reviewed in [21]). This pattern may be explained by "an intermittent filter barrier in the area of the Strait of Messina [which] probably controlled the processes and timing of the Late Middle Pleistocene-Late Glacial vertebrate faunal dispersal in Sicily" [Bonfiglio in: [22]]. Many Pleistocene fossil megafaunal elements entered Sicily from peninsular Italy [Bonfiglio in: [22]]. However, Bonfiglio [14] also hypothesized a Lower Pleistocene African origin of fossil elephants (*Elephas falconeri*) as a debatable alternative to derivation from European/Italian *E. antiquus *[Bonfiglio in: [22]]. Other paleontologists have also discussed whether Sicily had early Pleistocene connections to Africa, as in an, in this respect, unresolved investigation of a Pleistocene amphisbaenid lizard from Sicily [23]. All these authors [22-24], however, refer to the lack of conclusive evidence for a Pleistocene landbridge. Recent molecular data have suggested phylogeographic links across the Strait of Sicily, either based on very little data, or, with some speculation on the dating of these divergences [[20,25]; see Discussion].

### On green toads in the western Mediterranean region and North Africa

Green toads are widespread in the Palearctic region where they have differentiated into several lineages [26]. The occurrence of three bisexually reproducing ploidy levels [27] makes them a uniquely interesting vertebrate group. A recent survey of mtDNA variation characterized a deeply branched assemblage of at least twelve major haplotype groups [28]. Other data on green toad variation from North Africa have been relatively scarce [29,30]. Research has been mainly restricted to faunistics [31-33] or analyses of single populations (Egypt [34]) without taxonomic or phylogeographic focus. Green toad biology and ecology are relatively well known from the Balearic Islands [35,36], Corsica [[37] incl. refs.], Sardinia and mainland Italy [38]. In addition, morphometric analyses of green toads in Italy have been restricted to peninsular and Sardinian (plus Corsican) populations [38,39] until morphometric data for a population from northwestern Sicily became available [40]. Otherwise, regional research has been restricted to classical biogeography and faunistics including ecological and taxonomic remarks [41-43] and studies on phenology [40,44].

To gain insight into the phylogeography of the region, we test a wide range of possible substitution rates in order to better date the African-Sicilian divergence of green toads: Based on only two haplotypes from Sicily, Stöck et al. [28] roughly calibrated their tree by "assuming that the last landbridge between North Africa and Sicily broke about 5.3 Mya." Alternatively, this divergence could be younger. Here, we examine genetic data from many individuals and many more localities in Tunisia, Italy and Sicily, to scrutinize the North Africa-Sicily divergence employing a Bayesian coalescent demographic reconstruction method (BEAST 1.4.6). Using two mitochondrial and two nuclear sequence markers and additional morphological, phenological, bioacoustic and biogeographic data, we characterize green toads from Sicily as a separate evolutionary lineage that is a sister taxon to African green toads, but substantially different from other western Mediterranean green toad forms. These phylogeographic relationships substantiate scarce knowledge on western Mediterranean terrestrial biogeography and may have implications for comparative research on the phylogeography of other terrestrial animals in the region.

## Results

### Phylogeographic structure reveals a relationship across the Strait of Sicily

**Figure 2:**
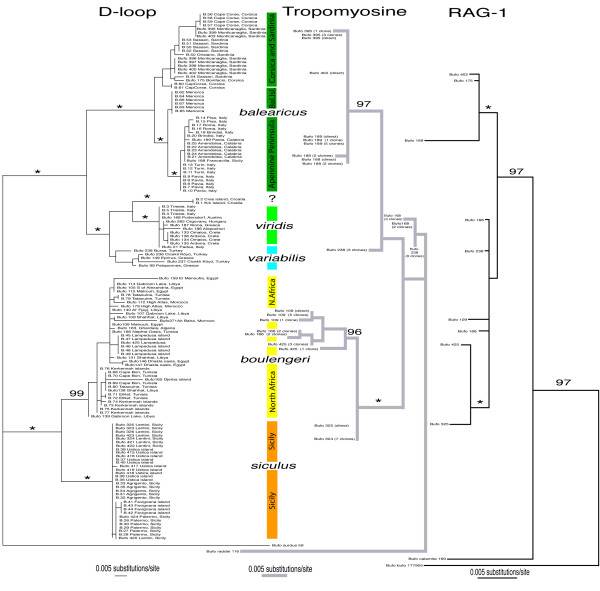
**Bayesian phylogenetic trees from the mitochondrial control region (d-loop; left), an intron of *alpha*-tropomyosine (right, gray) and a fragment of the RAG 1 gene**. The markers comprise 541 bp of the mtDNA control region, 405 bp of *alpha*-tropomyosine introns, and 860 bp of RAG-1; Bayesian posterior support values for major nodes are written above nodes, * indicates a value of 100% posterior probability. Each branch label in the d-loop tree contains the sample number and the major locality information. (Note that each label on the nuclear trees corresponds to the exactly horizontally opposite label of the d-loop tree. ("B." in the d-loop tree stands for *Bufo*; all samples labeled "B." were sequenced by A. Sicilia; all "Bufo" samples were sequenced by M. Stöck).

We found genetic markers in green toads (Additional file 1) within our geographic scope (Figure 1) to indicate five major spatially structured lineages (Figure 2). (I) The first lineage was found on Corsica, Sardinia, the Balearic Islands, Apennine Peninsula and the northeastern extreme of Sicily. This clade (*balearicus*) was different from other Eurasian mainland green toads (lineages II and III: *viridis *and *variabilis*), whose ranges border northeast to the Po River drainage and belong to widespread monophyletic groups in Eurasia [28]. (IV) Although geographically neighboring I, on most of Sicily and its surrounding islands another lineage (*Bufo *n. sp.) occurs that is substantially different from the first. (V) The entire North African range and the off-coast islands constitutes a fifth lineage (*boulengeri*). The details of the results defining each lineage are described below.

In particular, our analyses shows a deep sister relationship between African (V) and Sicilian (VI) groups and therefore a rarely studied phylogeographic connection across the Strait of Sicily.

### Characterization of the groups identified based on the Bayesian phylogram

Here we consider and name lineages that maintain their evolutionary integrity with respect to other lineages through both time and space and name them as species under the phylogenetic species concept.

#### Bufo balearicus Boettger, 1880

For details on taxonomy and nomenclature of *Bufo balearicus *[45] see supporting data in Additional file 2 (d). Analyses of mitochondrial 16S (not shown in tree) and control region sequences of 50 green toads from the Balearic Islands (loc. 5–7), Corsica (loc. 8–9), Sardinia (loc. 10–12), the entire Apennine Peninsula (loc. 13–16), Apulia (loc. 17), Calabria (loc. 18, 19), Marche (loc. 55, 56) and northeastern Sicily (loc. 20) form one very well-supported haplotype group (Bayesian posterior support: 100%; Figure 1, 2). Cloned tropomyosine intron alleles (Figure 2) from four representatives each from Sardinia (loc. 10), Calabria (loc. 18) and northeastern Sicily (loc. 20), as well as fragments of RAG-1 in three individuals each from Corsica (loc. 9), Sardinia (loc. 10), and Calabria (loc. 18) formed similarly highly supported clusters (Bayesian posterior support: 97% and 100%) and demonstrated that nuclear and mitochondrial markers show essentially the same signals.

**Figure 3:**
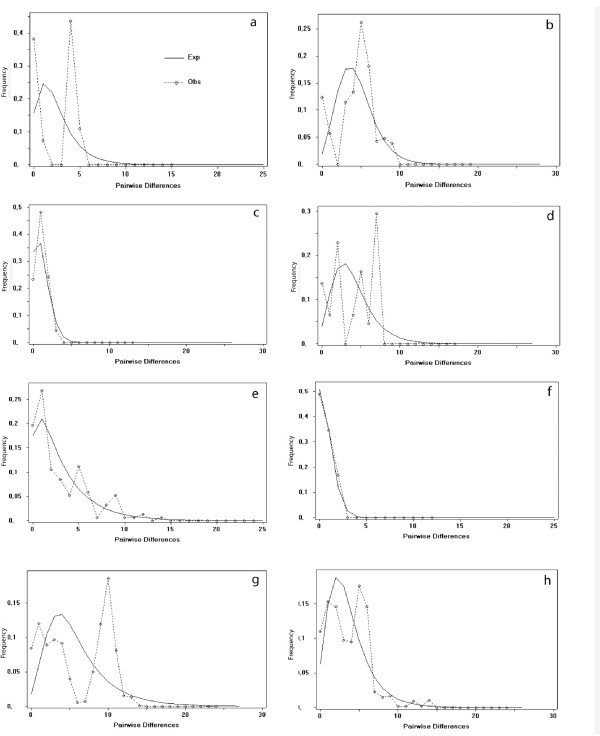
**Mismatch distributions from 541 bp of the mtDNA control region**. The dotted line shows the frequency distribution of the observed pairwise differences. The solid line shows the frequency distribution of the expected pairwise differences under the sudden expansion model, performed in DnaSP v. 4.10.9. Pairwise differences are counts of *i*, nucleotide differences; frequency is Fi [89] The model was applied within each group, and to each taxonomic group as a whole: a. *B. balearicus *from the Balearic Islands. b. *B. balearicus *from Corsica and Sardinia. c. *B. balearicus *from the Apennine Peninsula; d *. B. boulengeri *islands off the African coast. e. *B. boulengeri *from African mainland f. *B. siculus *n. sp. from Sicily g. all *B. balearicus *including Balearic Islands, Corsica, Sardinia, and Apennine Peninsula h. all *B. boulengeri *including African mainland and off-coast islands.

The control region tree also revealed three well-supported subclades: one comprising toads from Corsica and Sardinia which exhibit some geographic intermixing between genetically differentiated lineages within the islands; a second much-less structured clade including toads from the entire Apennine Peninsula from Turin, Pavia and Marche (loc. 13, 14, 55, 56) in the north to Apulia (loc. 17) and Calabria (loc. 19) in the far south; and a third subclade (nested between the two others) containing toads from the Balearic Islands. Subclade structure is evident in the average F_ST _values between each of these subclades (Table 1): Analyses of population structure confirmed high genetic differentiation (pairwise F_ST _= 0.7585, p = 0.00000) between Italian mainland and island toads (Corsica, Sardinia). Demographic analyses performed in *Fluctuate *estimated a greater than ten-fold exponential growth rate for the clade on the Apennine Peninsula than for the mitochondrial group on Corsica and Sardinia (767.1 > 51.7), suggesting population expansion on the mainland. However, log-likelihood tests did not reject a scenario of zero growth (Table 2). Growth of the mainland population was also not significantly supported by the mismatch distribution analysis in which the observed distribution matched an expected distribution for an expanding population; however, the shape and magnitude of the observed and expected distributions are very similar, suggesting some support for expansion on the mainland (Figure 3c). By contrast, this analysis, both graphical and statistical, supports stable populations on Corsica and Sardinia (Figure 3a, b). Tajima's D estimates for all *balearicus *groupings were not significant, and thus population expansion was not indicated by this test.

**Table 1:** Pairwise F_ST _between groups of West Mediterranean green toads based on sequences of the mitochondrial d-loop. All F_ST _-values are significant (p < 0.0000).

	*B. balearicus *Corsica+Sardinia	*B. balearicus *Balearic Islands	*B. balearicus *Apennine Peninsula	*B. boulengeri *Offshore Islands, N. Africa	*B. boulengeri *North African Mainland
*B. balearicus *Balearic Islands	0.60662				
*B. balearicus *Apennine Peninsula	0.75859	0.49501			
*B. boulengeri *Offshore Islands, N. Africa	0.91530	0.91812	0.95058		
*B. boulengeri *North African Mainland	0.77888	0.74269	0.82886	0.16073	
*B. siculus*	0.97543	0.98188	0.98815	0.96950	0.87081

**Table 2:** Estimates of historical demographic parameters in various green toad taxa and groups from mtDNA control region analyses using parameter estimation in the programs *Fluctuate *and DnaSP.

Mitochondrial control region clade	Taxon	N	Theta*	*g*^@^	Ln (like-lihood) for L max	ln (likely-hood) for zero growth	2(L_max_- L_g _= 0)	No growth can be rejected	MinAge estimate for corrected rate (pi-Net)	MinAge estimate for non-corrected rate (pi-betw.)	Tajima's D^#^
[All *B. boulengeri: *North Africa including islands]	*B. boulengeri*	37	0.0258	175.878	0.0041	-2.0458	4.0998	Yes	1.6 My	0.9 My	-0.66664
North Africa mainland	*B. boulengeri*	18	0.0377	263.037	0.1060	-3.5830	7.378	Yes	2.2 My	1.4 My	-1.63869
Sicily and adjacent islands	*B. siculus *n. sp.	33	0. 0015	2585.02	0.6440	0.7064	-0.1248	No	NA	NA	0.74861
[All *B. balearicus: *Apennine Peninsula, Corsica, Sardinia, Balearic islands]	*B. balearicus*.	61	0.0116	184.754	0.5139	0.1018	0.8242	No	NA	NA	0.74773
Corsica, Sardinia	*B. balearicus*.	21	0.0177	51.788	0.0455	-0.1783	-0.447	No	NA	NA	-0.07834
Apennine Peninsula	*B. balearicus*.	29	0.0029	767.136	0.0364	-0.2254	0.5236	No	NA	NA	0.19720

* = 2N_e_μ estimate of the distribution of coalescence times in the population at the time the sample was taken, estimated by *Fluctuate*

@ = exponential growth parameter estimated by *Fluctuate*.

# all values non-significant

The Bayesian phylogenetic analysis shows *B. balearicus *to be reciprocally monophyletic on the mitochondrial level, albeit using a subsample of individuals within each group, with respect to all other green toads; thus our data corroborate the proposal that *B. balearicus *be given the status of a separate species (see Additional file 2 for details on nomenclature).

#### Bufo boulengeri Lataste, 1879 [46]

While exhibiting some internal structure, all North African control region (Figure 2) and 16S mitochondrial sequences (Additional file 1) of toads between the Atlantic coast of Morocco (loc. 1–3) and the Nile Valley of Egypt (loc. 37–40), including the off-coast islands of Kerkennah (loc. 30, 31), Djerba (loc. 32) and Lampedusa (loc. 26), form a well-supported monophyletic cluster (posterior probability = 99%) that is a sister clade to mitochondrial sequences of toads from Sicily (loc. 21–25), but that differs from toads from the Middle East [28]. Mitochondrial sequences show moderate differentiation between all islands considered together (Djerba, Lampedusa, and Kerkennah) and North African mainland haplotypes (Table 1: pairwise F_ST _= 0.16073, p = 0.00000), suggesting recent faunal exchange. *Fluctuate *suggested population growth for the green toads on the African mainland, as well as for all *boulengeri *considered together; log likelihood tests rejected no growth scenarios for both groupings (Table 2). Tajima's D calculated for the mainland group indicated some support for population expansion (-1.63869, 0.10 > p > 0.05); however, the mismatch distribution analysis did not support a growth model.

Tropomyosine intron sequences in African samples from Libya (loc. 36), Tunisia (loc. 29) and Lampedusa (loc. 26) exhibit the same tree topology as the mitochondrial markers and constitute a highly supported clade (posterior probability = 96%) that differs substantially from tropomyosine sequences from two Sicilian green toads (loc. 21). The very conserved RAG-1 marker shows two African samples in a polytomy with Eurasian green toads, from which both *B. balearicus *and two samples each from Sicily and Lampedusa differ.

#### Bufo n. sp

This taxon was identified on most of Sicily (loc. 21-23a) and two close islands (Favignana, loc. 25; Ustica, loc. 24). Demographic analyses performed in *Fluctuate *estimated θ to be an order of magnitude lower than that estimated for all other green toad groups examined except the mainland *balearicus *group (Table 2). Although the log likelihood test did not permit rejection of a no growth scenario, the very low initial θ estimated by the analysis indicates that few individuals may have founded the lineage. Similarly, the observed mismatch distribution is not statistically identical to the expected distribution under a sudden growth model (p = 0.077). However, the unimodal peak is shifted to the left of the distribution and very tightly matches the expected distribution for a recently expanded population (Figure 3f). Finally, Tajima's D did not indicate deviation from neutrality, and thus a scenario of population expansion cannot be invoked with this measure.

*Bufo *n.sp. exhibits reciprocal monophyly for both mitochondrial markers (and using the tropomyosine intron marker on a subsample of individuals) with all other groups/taxa (Figure 2). The RAG-1 phylogram shows toads from Sicily and Lampedusa forming a well-supported (posterior support = 100%) clade that differs from all sampled African and Eurasian green toads (Figure 2). For all mitochondrial and nuclear markers, *Bufo *n.sp. shows a much greater genetic distance from Italian mainland *B. balearicus *than from *B. boulengeri*, which inhabits all of North Africa. Taken together, as for the two preceding taxa, phylogenetic divergence of *Bufo *n. sp. is evident and we acknowledge this by describing it as new species (*Bufo siculus*, see below).

#### Bufo viridis (Laurenti, 1768) and old isolates

The haplotype group representing this taxon, as previously shown by Stöck *et al*. [28], was found exclusively in the very northeastern part of Italy, northeast of the Po River (loc. 41, 42). So far, two very well supported monophyletic and geographically widespread mtDNA groups of green toads are known from the Balkan region. The haplotype group dominating Asia Minor (*B. variabilis*) and that of *B. viridis *(Figure 1: blue and light green; [28]) apparently co-occur in Greece. However, neither of the two was found off the Adriatic coast on the Croatian islands of Krk and Cres (loc. 43, 44). Instead, a very well supported separate clade (posterior support = 100%) formed by two sequences of toads from those islands revealed an apparent relict group, which is more closely related to *B. viridis *than to *B. variabilis *(Figure 2), but further data are required to confirm this relationship.

### Age of African-Sicilian vicariant separation

Divergence time estimates for the principal mitochondrial (control region and 16S rRNA) clades recovered are shown in Table 3. We provide the 95% highest posterior density intervals (95% HPD, that is, the shortest intervals that include 95% of the posterior sampled values) as well as the mean of the sampled values. Time estimates are based on the reconstruction of the most common recent ancestor for the mtDNA control regions under the coalescent (see Material & Methods section for details). The 95% HPD, although overlapping, show consistent values within and between clades. Divergence between African and Sicilian haplotypes falls within the range of 0.6 and 3.5 My [mean 1.8 My, around the Pliocene/Pleistocene boundary (~1.8 My)] for the control region. Divergence time estimates were also derived using the 16S data (see Material & Methods for details). Results were similar to those obtained using the mtDNA control region (divergence between African and Sicilian haplotypes: 95% HPD from 0.164 to 3.603 My (mean: 2.051); split of *balearicus*/*viridis *clades: 95% HPD from 0.18 and 3.795 (mean: 2.123). All the dates estimated in our analysis seem to represent post-Messinian divergence events.

**Table 3:** Within-clade (numbers in *italics*) and between-clade (numbers in regular font) divergence time estimates obtained using a coalescent-Bayesian framework as implemented in BEAST v1.4.6 applied to the mitochondrial control region and 16S sequences (not available for *B. variabilis*). The African-Sicilian divergence time estimates (*boulengeri *to *siculus*) are printed in **bold**. Estimates are shown in My. Values in parentheses show the 95% highest posterior density intervals (95% HPD); they represent the shortest intervals that contain 95% of the posterior sampled values.

*MtDNA clade(s)*	Divergence time estimate	*MtDNA clade(s) *and/or Geographic region	
			
	Control region 16S		
*boulengeri*	*1.2 (0.394–2.351)* *1.57 (0.099–2.80)*	*boulengeri*	N-Africa
*siculus*	*0.677 (0.115–1.53)* *1.56 (0.101–2.69)*	*siculus*	Sicily
*balearicus*	*0.914 (0.227–1.875)* *1.84 (0.15–3.365)*	*balearicus*	Apennine Peninsula, Corsica, Sardinia, Balearic Islands
*variabilis*	*0.85 (0.172–1.838)*	*variabilis*	Balkan, Anatolia
*viridis*	*0.527 (0.096–1.152)* *1.44 (0.196–2.785)*	*viridis*	Central, E-Europe
*balearicus-variabilis-viridis*	*1.956 (0.66–3.795)*	*balearicus-variabilis-viridis*	
*boulengeri*	**1.833 (0.635–3.509)** **2.05 (0.16–43.60)**	*siculus*	
*boulengeri-siculus*	2.749 (1.188–4.906)	*balearicus-viridis-variabilis*	

### Multivariate morphometric comparisons

**Figure 4:**
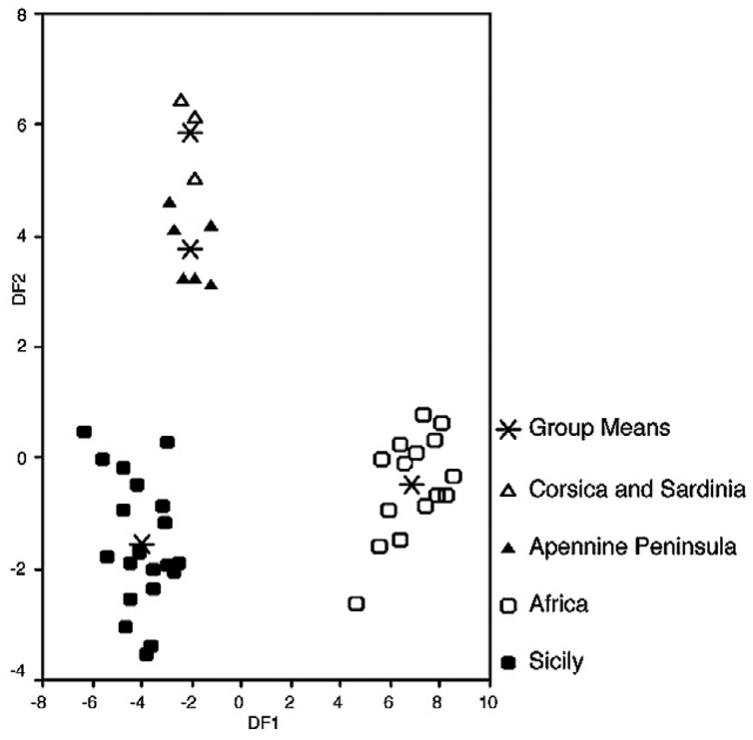
**Result of a discriminant analysis with 20 morphometric traits**. DF1: Discriminant function 1, DF2: Discriminant function 2 on four groups of green toads specified in the legend.

A discriminant analysis using all 20 characters demonstrated the distinctness of toads from Sicily by correct reclassification of 100% of individuals into the four groups from: (i) Sicily, (ii) N-Africa, (iii) Corsica and Sardinia, and (iv) the Apennine Peninsula. Although encoded as belonging to different geographic groups, the genetically more closely-related groups (iii) and (iv) of the *B. balearicus *clade showed fewer differences (Figure 4), making us confident of the power of the analyses' and this morphometric dataset. However, in order to test for potential over-parameterization of the analysis, we also reduced the number of morphometric characters to five, which again reclassified 100% of the individuals into their four respective groups.

## Discussion

### A phylogeographic relationship between Africa and Sicily

We have demonstrated a sister relationship on the mitochondrial and nuclear levels between green toads from Sicily (*B. siculus*, see below) and those from the entire North African range (*B. boulengeri*). The phylogenetic depth of this divergence, which may range between 0.63 My and 3.5 My (mean 1.83 My) for the control region and from 0.164 to 3.603 My (mean 2.051) for the 16S rRNA, probably excludes human introduction and reveals an infrequently-studied phylogeographic relationship in terrestrial vertebrates. This evolutionary connection may be significant for the phylogeography of the Mediterranean and have implications for research on other terrestrial animals. While some biogeographic studies have suggested common ranges for terrestrial vertebrates (for example the "Siculo-Maltese-Maghrebin" range type of Turrisi and Vaccarro [42]), and paleontologists discuss a possible early Pleistocene faunal exchange with North Africa (see introduction), so far, very few molecular studies have suggested any genetic relationships within terrestrial vertebrates across the Strait of Sicily. Two examples come from species (e.g. *Discoglossus *[25]; *Chalcides *[47,20]), for which human introduction elsewhere at circum-Mediterranean sites has been demonstrated and for which African-Sicilian relationships have never been demonstrated using any nuclear sequence data. Despite some recent speculation (e.g. *Crocidura sicula*, potential Messinian origin from North Africa [16]), a possible African origin of terrestrial Sicilian fauna has rarely been thoroughly tested with molecular methods, and never using any nuclear sequence, and thus supported by both mitochondrial and nuclear sequence data. To our knowledge, a genetic African-Sicilian link has only been inferred in two other terrestrial species, but even these have ambiguous information on timing and direction: (i) Phylogenetic inference based on allozymes regarding relationships among *Chalcides *lizards from Sicily, Africa, the Apennine Peninsula and Sardinia [47] showed Sicilian skinks most closely related to Italian mainland skinks (Calabria in the south to Liguria in the north), while lizards from Tunisia appeared genetically almost identical to Sardinian *Chalcides*, interpreted as a human introduction from Tunisia into Sardinia. Very recently, however, Giovannotti et al. [20] found two Sicilian *Chalcides *mtDNA haplotypes to have a sister relationship with Tunisian and Sardinian haplotypes (the latter data not shown in the *Chalcides *phylogeny) and interpreted this as a possible Lower Pleistocene (1.8 My) colonization event of Sicily (and Italy) from North Africa. (ii) Using allozymes and cytochrome *b*, Zangari et al. [25] assessed lineage relationships among discoglossid frogs from multiple locations in the western Mediterranean (including Sicily, Sardinia, Tunisia). These authors showed a deep divergence of some mtDNA haplotypes across the Strait of Sicily, but also some nearly identical haplotypes across Sicily, Tunisia and Algeria. While the authors commented imprecisely (i.e., they did not specify if the following referred to Gibraltar or Sicily or both) that the "spread of *Discoglossus *between Europe and Africa should have occurred at the end of the Messinian salinity crisis", they interpreted closely-related haplotypes across the Strait of Sicily as "little genetic differentiation detected among Algerian and Tunisian *D. auritus *with respect to Maltese and Sicilian *D. pictus *suggest [ing] a very recent isolation", without further interpreting or dating their results. The significance of land connections for faunal exchanges between Africa and Sicily is mildly challenged by the well-known salt tolerance of green toads. Gordon [48] experimentally demonstrated survival of *B. viridis *in seawater for several hours or days. Breeding and swimming in brackish (beach or desert) pools and waters [49] as well as estuaries has been observed [38]. Therefore, green toads may have the potential for transmarine dispersal. The distance from Africa to Sicily (~140 km), however, can be considered a very strong barrier, even during low sea levels (>45 km). In addition, external fertilization (and absence of brood care) requires that several adults or larvae disperse in order to found new populations. This and the early divergence time (0.63 My < 1.83 My < 3.5 My) of African and Sicilian lineages make it more plausible that toads with a terrestrial life style dispersed via land connections than by rafting. This hypothesis was supported in the recent study of terrestrial *Chalcides *lizards (see above), which estimated a similar divergence between Tunisian and Sicilian clades (1.8 My [20]) to the one we propose for green toads. However, several papers have suggested the ability of anurans to overcome large sea barriers [50,51], especially in the tropics, where swimming islands may facilitate rafting. Therefore, comparative phylogeographic data from other terrestrial species in Sicily and North Africa would be necessary to test the feasibility of competing hypotheses.

Divergence time estimates based on molecular data normally rely on external calibration points from the fossil record or from well-known paleogeographic events. Because neither fossil nor paleogeographic calibration points were available, we calculated divergence times using an uncorrelated relaxed molecular clock and a range of substitution rates for the two mitochondrial fragments. We acknowledge a potential limitation, that is, the use of only mtDNA to determine divergence times, which may lead to overestimation of the splitting dates. This overestimation may occur because the most recent common ancestor (MRCA) of the haplotypes (their coalescent) does not necessarily correspond to the real temporal split of the populations but may precede the actual divergence of the populations [52]. We consider substitution rates between 1% and 3% per lineage per My to be reasonable values for amphibian mtDNA (for references see Materials and Methods) although they have not been empirically assessed in green toads. When regions with highly repetitive motifs are absent, as in green toads, the control region tends to show higher rates of substitution than the rest of the mitochondrial molecule [53,54]. Thus, the substitution rates selected here are conservative, higher than the rate for the rest of the mitochondrial molecule, but lower than the fastest observed rates for the control region in other taxa. Higher rates would imply younger dates for the splitting of the African and Sicilian lineages. This point is illustrated by our temporal estimates from the mitochondrial 16S gene which we obtained applying a slower substitution rate, which ranged from 0.03–1% per lineage per My (see refs. in the Discussion). The two markers, with different substitution rates, yield similar 95% HPD time intervals.

Although the values reported should be regarded with the caveats mentioned, it is highly improbable that the vicariant event that separated the African and Sicilian green toads took place during the Messinian (5.3 Mya), that is, earlier than the range of dates estimated by this method. Instead, it is very likely that the Africa-Sicily divergence is post-Messinian. In order to validate the hypothesis proposed here and to test competing paleobiogeographic scenarios, a formal comparative phylogeographic study including more genetic markers and other terrestrial species in Sicily and North Africa would be necessary. Several new comparative phylogeographic approaches have been proposed [55-57], each requiring the inclusion of more data for a more reliable statistical inference to be obtained.

### Historical biogeography of *Bufo balearicus*

The second major result of our study is that green toads from most of the Apennine Peninsula, Corsica, Sardinia and the Balearic Islands represent a separate taxon, which is different from other Eurasian green toads on the mitochondrial and nuclear levels. Three *B. balearicus *subclades emerged from our mtDNA control region data; the subclade on Corsica and Sardinia exhibited basal haplotypes and the most substantial structure. These observations suggest speciation of *B. balearicus *on these islands around 0.9 Mya to 1.8 Mya (Table 3). The earliest green toad fossils in Italy are known from the Late Miocene from the northeast (Ravenna Province) and from the Pliocene in the southeast (Gargano region) of the Apennine Peninsula [58]. They have also been identified from the Pleistocene of Corsica and Sardinia [59]. Corsica and Sardinia had landbridges to (or were separated only by narrow marine straits from) the Apennine Peninsula during several geological periods (~18 Mya; 9 Mya; 5.3 Mya [60]). Although a narrow strait may have mostly separated Corsica/Sardinia from Tuscany [61], paleontological [62,63] and phylogeographic data (*Discoglossus *[25], isopods [64]) suggest limited early Pleistocene faunal (perhaps oversea) exchange between these islands and the mainland.

This same signature is suggested by our data. The *balearicus *clade widespread on the Apennine Peninsula displayed a mismatch distribution visually, but not statistically fitting that expected for a population that underwent a sudden expansion (Figure 3). Similarly, the coalescent simulation program *Fluctuate *estimated a growth parameter ten times higher for the peninsular group than for the island *balearicus *clade (Table 2), suggesting recent expansion, perhaps after its arrival on the mainland; however, a no growth scenario could not be rejected statistically. Tajima's D did not support a demographic expansion in either of the *balearicus *groups separately, or combined. The northeastern range limit of *B. balearicus *appears to be the Po River drainage, which may also be the southwestern boundary for *B. viridis*. Recent mapping of green toad distributions in Italy [38], however, shows an apparently continuous range from the northeast (probable haplotype group of *B. v. viridis*) to the southwest (haplotype group of *B. balearicus*) across the Po drainage. It is likely that the groups are in contact in that area; discerning contacts and possible hybridization dynamics represent challenging future research topics. Indeed, the Po drainage seems to be a biogeographic border and/or contact zone between variously related taxa of amphibians and reptiles (for overview [65]: *Rana latastii/R. italica*; *Hyla arborea*/*H. intermedia *[66]; *Bombina variegata/B. pachypus *[67]; *Rana lessonae*/*R. bergeri*[68]) or, it is considered a "source of genetic variability" [69].

Fossils demonstrate the presence of green toads on the Balearic Islands from the Upper Pleistocene of Mallorca [70], but early human introduction from Corsica was proposed as the source of green toads on these islands [35]. Our data show toads from Mallorca and Menorca to be nested within the mitochondrial clades from the Apennine Peninsula and Corsica/Sardinia, and thus corroborate serum albumin data on toads from Mallorca and Corsica that prove them more closely related to each other than to green toads from Palestine, Africa, and Greece [35]. We cannot locate the exact geographic origin of Balearic green toads with our current data; faster evolving nuclear markers and denser sampling in regions of possible origin are required. Our data also corroborate provisional allozyme data (Lattes, A. 1997, Abstr. 3rd World Congr. Herpetol., Prague: p. 123) that associated toads from Corsica, Sardinia and NW-Italy (all from the *B. balearicus *range) with a ~0.25 Nei's distance from toads from Vienna based on a UPGMA dendrogram (*B. viridis*; [71] quoting Lattes' abstr.).

Although Calabria consisted of islands until the Pleistocene [72], episodic Upper Pleistocene faunal exchange across the Strait of Messina is well documented by the fossil record [72]. While few amphibians crossed the Strait of Messina [67], *B. balearicus *haplotypes have been detected in northeastern Sicily (loc. 20).

### Biogeography of *Bufo siculus *n. sp

Our phylogeographic findings agree with paleogeographic and fossil data [72], which suggest a long Plio-Pleistocene isolation of Sicily. After their "out of Africa" origin, green toads apparently spread across most of Sicily, where they are well known from the Pleistocene fossil record [22]. So far, no phylogeographic signature of the Pliocene subdivision of Sicily into two islands was detected. The effective population size of mtDNA in *Bufo siculus *n.sp., as inferred from θ (*Fluctuate*) is small (Table 2).

The distribution analysis indicated an observed distribution in the *B. siculus *clade that was unimodal and visually congruent with the distribution of expected values for a sudden population expansion, although not quite statistically significant (Figure 3f, p = 0.077). The distribution for this clade is extremely compressed to the left, indicating very low numbers of mismatches and low variance. This profile may indicate an even more recent expansion in this group than in the mainland *B. balearicus *group. The *Fluctuate *analysis also did not statistically permit rejection of a no-growth scenario for this clade, and Tajima's D did not fit the expectation for an expanded population.

However, the high similarity among mtDNA haplotypes across the entire island is consistent with the known high mobility of green toads, exemplified by fast post-Pleistocene re-colonization of northern Europe by two haplotype groups [28]. The current range of *B. siculus *appears to follow a pattern known in another Sicilian endemic, the lizard *Podarcis wagleriana*, that is widespread across Sicily but absent in the very northeast [42,73].

Our data reveal taxonomic identity (*B. siculus*) of toads on Favignana Island (loc. 25) and Ustica Island (loc. 24); green toads on the Aeolian Islands may be members of the geographically proximate *B. balearicus *(see Additional file 3 for further comments on Circum-Sicilian islands). Detection of Calabrian *B. balearicus *haplotypes in northeastern Sicily (loc. 20) suggests their relatively recent invasion; either during Pleistocene sea level lows [72], by rafting across the narrow Strait of Messina, or possibly by human introduction. It remains unknown whether both taxa (*B. balearicus *and *B. siculus*) occur in sympatry in northern Sicily as range maps suggest [42], and whether they would hybridize, given the considerable mtDNA and breeding-phenology differences. Bioacoustic data in the potential contact zone will also help to illuminate this question.

### Description of a new species

Mitochondrial and nuclear, morphometric and other preliminary biological data show that green toads from most of Sicily represent a separate lineage. Sicilian green toads have been physically separated by the Strait of Sicily from their closest African relatives for long evolutionary periods. In order to acknowledge these facts, and to raise the potential conservation status of this form, which represents an island endemic, we hereby describe it as a new species.

### *Bufo siculus *n. sp

#### Holotype

**Figure 5:**
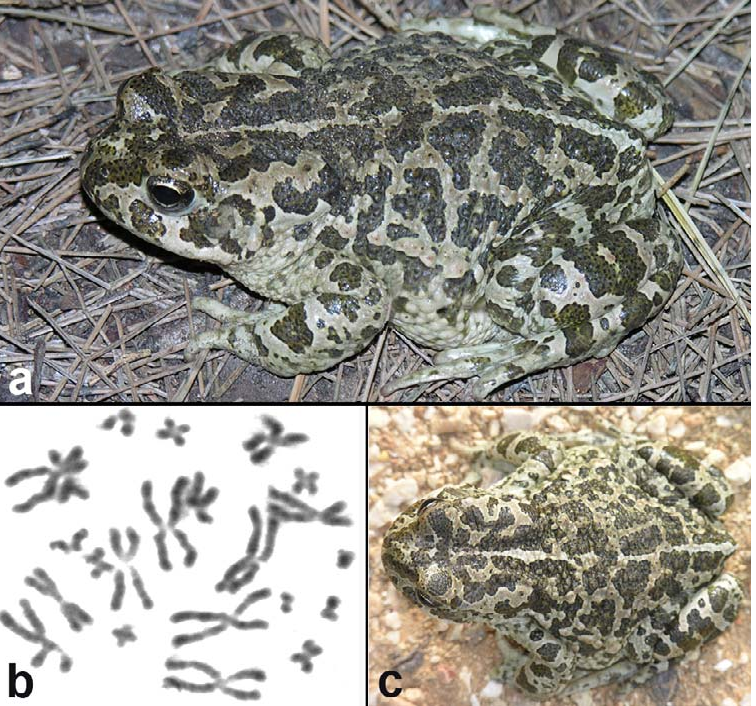
**Holotype of *Bufo siculus *n.sp. and chromosomes of the new species**. (a, c) Holotype of *Bufo siculus *n.sp. (P215, Museum of Terrasini, Palermo), adult female, in life (Photo: M. Lo Valvo). (b) Somatic metaphase from a paratype (MVZ 250742), southeastern Sicily, San Leonardo River, with 2n = 22 chromosomes (Photo: M. Stöck).

An adult female, P215 (Museum of Terrasini, Palermo, Figure 5a, c), collected November, 17, 2006, by M. Lo Valvo, Monte Pellegrino reserve (38.170 N, 13.351 E), Palermo, Sicily, Italy; for nomenclature and morphological description: Additional files 2 and 4.

#### Paratypes

ZFMK 85896 (juv., topotypic, M. Lo Valvo leg. 2005), ZFMK 85778 (ad. male, Sicily, Monte Carbonara region, W. Haas leg., ca. 1991, GenBank EU497506), ZMB 69556 (juv. female, EU497501) and MVZ 250741 to MVZ 250743 (two juv. and one subad. female, EU497502 to EU497504): Italy, southeastern Sicily, mouth of San Leonardo River, M. Lo Valvo leg. 2005; MZPA A95 (adult female, La Fossa, NW Sicily, A. Sicilia leg. 2006, EU497598); NME A 1490/08 (adult male, La Fossa, NW Sicily, F. Marrone leg.). For morphometric details of two adult paratypes see Table 5 in Additional file 4.

#### Diagnosis

A medium- to large-sized green toad that differs from all other, especially all circum-Mediterranean green toad species, by its distinct mitochondrial haplotype group. *Bufo siculus *exhibits strong variability in coloration with adult males showing less contrast in marbled patterns than females. Brownish to olive (but barely bright greenish) spots often form light dorsal stripes (not to be confused with a yellowish pigmented stripe), a character rarely found in other green toads of Italy, but which is quite common in *B. boulengeri *from North Africa. The new species is distinguishable from its geographic neighbor,*B. balearicus *(as defined by mtDNA haplotype group, Figure 1, 2), which exhibits pinhead-sized red (female *B. balearicus*) or brownish (male *B. balearicus*) spots around tips of lateral glands, by the lack of these spots. *Bufo siculus *almost never shows a reddish-orange coloration, characteristic of many *B. balearicus*, the only form of the subgroup with which it may co-occur on Sicily. In Sicily, the ratio VDT/ED [vertical diameter of the tympanum (VDT) divided by the diameter of the eye (ED)] is smaller in *B. siculus *[0.530 (max) ≥ 0.381 (mean) ≤ 0.247 (min) (N = 42)] than in allo- or parapatric *B. balearicus *[0.622 ≥ 0.478 ≤ 0.345 (N = 31)], but similar to that of African *B. boulengeri *[0.527 ≥ 0.362 ≤ 0.25 (N = 29)]. In life, *B. siculus *has a dark yellowish-golden iris.

#### Variation

The morphometric variation of topotypic *B. siculus *with a focus on sexual dimorphism has been recently studied by Lo Valvo & Giacalone [40] in 354 males and 312 females. In other parts of its range, stronger variability in size occurs.

#### Karyotype

Diploid, 2n = 22 chromosomes; a mitotic metaphase is shown in Figure 5b.

#### Advertisement calls

**Figure 6:**
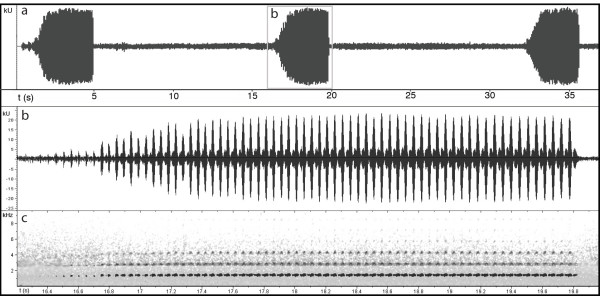
**Oscillogram and spectrogram of an advertisement call of a male *Bufo siculus***. Recorded at 16°C at the type locality (recorder "Marantz CP430" with Philips unidirectional electric condenser microphone). a. Oscillogram of a call train of ca. 35 s with three calls. b. Zoomed part of the framed (b) section (in Figure 6a) between 16.2 s to 19.95 s (note slight overlay with another male calling in the background). c. Spectrogram of the call section shown in b. The fundamental frequency is at around 1600 Hz and two harmonic vibrations are visible.

An advertisement call from the type locality (Figure 6, recorded as described [75] at 16°C) shows a green toad trill (similar to *B. viridis*; reminiscent of a canary trill) consisting of a series of up to 75 actively pulsed notes with a constant duration separated by constant internote intervals (Figure 6b, c). Single notes show a symmetrical amplitude increase and decrease as typical of actively pulsed calls in Palearctic green toads. Note series are interrupted by ("inter-call") intervals of more than 12 s (at 16°C, Figure 6a). Sonagram and oscillogram (Figure 6) reveal deviations in pulse structure at the beginning of the call, with increasing frequency and signal intensity, as is typical of the subgroup [75]. The fundamental frequency of the advertisement call, from the topotypic male shown in Figure 6, was 1600 Hz. The advertisement calls are similar in their note repetition rate ("pulse rate") to and typical of other diploid members of this complex [75,76] but deserve further investigation with higher sample size across a range of temperatures.

#### Distribution

Figure 1 and this paper.

#### Breeding phenology

Breeding phenology may be explained by phylogenetic history. Lo Valvo & Giacalone [74], and Sicilia et al. [44] reported differences between Italian mainland (including Calabrian, i.e.*B. balearicus*) and Sicilian green toads: *B. siculus *exhibits a much longer, potentially bimodal breeding period (January-June and September-November), and high plasticity similar to African *B. boulengeri *(scarce data discussed in [44]), versus a short reproductive period in spring (February-April) that is typical of *B. balearicus*.

## Conclusion

Our findings on green toads give the first combined mitochondrial and nuclear sequence evidence for a phylogeographic connection in terrestrial vertebrates across the Strait of Sicily. Given the available literature on substitution rates of the anuran d-loop and 16S rRNA, which range conservatively from 1% to 3% to higher rates [53,77] and from 0.3% to 1% [78,79], respectively, we argue that the African-Sicilian divergence of green toads may be younger (but not older) than estimated by the present analyses. This points towards a post-Messinian (< 5.3 My) faunal exchange between Africa and Sicily. This relationship may have implications for comparative phylogeographic research on other terrestrial animals that co-occur in North Africa and Sicily. We highlight the necessity for similar phylogeographic studies that use more molecular markers to provide accurate estimates of the potential vicariant events and barriers, as well as the precise dating of the splits between the lineages involved.

## Methods

### Sequencing of mitochondrial and nuclear DNA

Genomic DNA was extracted from frozen or ethanol preserved blood, liver, muscle tissue, tail tips (tadpoles) and muscle of vouchers from scientific collections using the Qiagen DNeasy™ kit. We either amplified most of the mitochondrial control region (or "d-loop", ~860 bps [28]) or a shorter fragment (577 bps) of the control region using the primer pairs CytbA-L/ControlK-H (PCR: 95°C, 3 min, denaturation; cycle [94°C, 45 s, denaturation; 55°C, 45 s, annealing; 72°C, 60 s, extension] 35 times; 72°C, 5 min, final extension [28]).

In those individuals from which we sampled the shorter control region fragment, an additional 611 bps of mitochondrial 16S rRNA were amplified using the primer pairs 16Sar-L/16Sbr-H (PCR: 95°C, 3 min, denaturation; cycle [94°C, 45 s, denaturation; 55°C, 45 s, annealing; 72°C, 60 s, extension] 35 times; 72°C, 5 min, final extension [80]).

In representatives from all major mitochondrial clades, we sequenced two nuclear markers. To amplify a fragment of ~880 bps of RAG-1 (recombination activating gene), we used the primers MartFL1 and AmpR1 [81] in a touch down PCR (95°C, 4 min, denaturation; first cycle [95°C, 30s; decreasing annealing temperature from 60°C to 45°C of -1°C per cycle, 30 s; 72°C, 1:30 min] 15 times; followed by a second cycle [95°C, 30s; 45°C, 30 s; 72°C, 1:00 min] 20 times; 72°C, 10 min). We also applied primers developed by Friesen et al. [82] for birds to amplify an intron of *alpha*-tropomyosine, situated between the exons 5 and 6, for the first time (to our knowledge) to anuran amphibians. PCR conditions for Friesen's primers proposed for "frogs" were adapted as follows: 95°C, 1:30 min; cycle [94°C, 30 s; 55.9°, 30 s; 72°C, 45 s] 30 times; 72°C, 5 min. All PCR products were sequenced directly and apparent heterozygote genotypes of tropomyosine were also cloned using the pGEM^®^-T vector system (Promega). PCR product concentrations were quantified (NanoDrop^® ^ND-1000 spectrometer) and adjusted to 25 ng/μl. We mixed 1.5 μl template, 0.075 μl of vector (50 ng/μl), 2.5 μl 2× ligation buffer, 0.5 μl T4 ligase, and 0.425 μl water and ligated overnight (10°C). Transformations (2.5 μl ligation plus 12–25 μl competent cells) were recovered in SOC for 1 h 30 min; 80–100 μl of cell suspension was applied to small agar plates. After incubation (18 h, 37°C), at least eight white colonies were amplified with vector primers M13forw./M13rev. Nested vector primers T7 and Sp6 (Promega) were used as sequencing primers.

All PCR-products and clones were sequenced in both directions and visualized on an ABI 3730 sequencer. Sequences were aligned using Sequencher, v. 4.1.2 and edited using MacClade 4.06.

### Phylogenetic analyses

For each sequence fragment, the best fitting model of sequence evolution was selected using MrModeltest [83]. Phylogeny was inferred for each locus separately, using the program MrBayes (v. 3.0b4 [84]), running four chains for 5 or 10 million generations, with tree sampling every 1000 generations. The "burnin"-value was selected by visualizing the log likelihoods associated with the posterior distribution of trees in the program Tracer. All trees generated before the log likelihood curve flattened out were discarded. From the two different control region fragments, we assembled an overlapping alignment of sequences comprising 541 characters from all 148 individuals included in this study; the best fitting model of evolution was HKY+G (AIC; 10 M generations; burnin = 1500, the same settings were used for the 16S rRNA sequences). For RAG-1 fragments (860 bp, model: GTR+G; AIC, burnin = 1000) and the *alpha*-tropomyosine intron (612 bp; model: HKY+G; AIC; because of the occurrence of insertions and deletions, all missing and ambiguous data were excluded and only 405 bp were analyzed; 5 M generations; burnin = 1000).

Because amplification or alignment of markers was not equally possible for identical outgroup taxa from the available material, more than one species had to serve as outgroup. In previous analyses [[28] and unpublished data], we demonstrated that all used taxa (*Bufo surdus, B. raddei, B. calamita, B. bufo*) represent suitable outgroup species.

### Taxonomic subdivision

To evaluate the distinctiveness of genetic groups of West-Mediterranean green toads of the *B. viridis *subgroup [sensu [28]], and of further geographic subdivisions within each genetic group, we calculated pairwise F_ST _values between all groups using Arlequin 2.0 [85] based on the mitochondrial control region.

### Demographic analyses and divergence time estimates

We assessed the possibility of population expansion for green toad groupings assembled into different geographical populations. We applied three different tests, each with different strengths, to the mitochondrial control region dataset (846 or 541 bp), to detect evidence of recent expansion. First, we applied *Fluctuate *[86], a maximum-likelihood estimator of the parameters θ and g (θ = 2Ne/μ; g = exponential population growth rate parameter). The exponential growth parameter (g) was used to estimate the size of the population at time in the past from N_t _= θ^e-(gμ)*t *^where N_t _is the effective population size at time *t *in the past [86]. Using this equation, *t *was estimated by substituting N_t _with N_t_/N_t = 0 _= 0.1 (μ is DNA substitution rate per site per generation, N_t _is the female effective population size at time t). Repeated analyses to ensure stability of estimates were run as described by Stöck *et al*. [28]. Growth was inferred using logarithmic likelihood ratio tests with one degree of freedom [87].

Second, DnaSP version 4.0 [88] was used to calculate and show in graphic form the distributions of observed and expected pairwise nucleotide site differences, also called mismatch distributions, between all individuals within each group, and the respective expected values for growing populations [89]. The model of sudden expansion describes an initial population at equilibrium, with the expected pairwise differences, θ_*0*_, (θ = 2N_e_μ) and assumes rapid population growth, resulting in θ_1 _(Theta final). Tau, τ, is the time of the growth measured in units of substitutional time (τ = 2μt; t is the time in generations, μ the substitution rate per locus and per generation [89]). Graphically, the mismatch distribution of a recently expanded population is unimodal and smooth; the wave-shaped curve is centered nearer the y-axis the more recent the expansion, moving away as the number of mismatches increases [89]. By letting θ_1 _become very large, θ_*0 *_and τ are estimated from the data [90]. We set θ_1 _to 1,000,000. We assessed the deviation of the observed distribution from the expected under a model of sudden expansion by comparing the raggedness statistics of the observed distribution with a simulated distribution to determine the probability that the raggedness of the observed distribution could have arisen by chance. Third, we estimated Tajima's D [91] in DnaSP for each grouping. Tajima's test of selective neutrality compares two θ estimators and its significance is evaluated by comparison of the test statistic (D) with values randomly generated under "neutrality." Significant values indicate the population has deviated from neutrality, or that another demographic force has caused the deviation from expectation, such as population expansion (significant negative D).

Divergence times among the main mitochondrial lineages were estimated using a Bayesian-coalescence approach, as implemented in BEAST 1.4.6 [92-95]. In analysis of the control region, we used a matrix of 60 individuals and 752 bp. We started the search with an UPGMA tree, constraining the clade *B. boulengeri*-*B. siculus *from Sicily to be monophyletic. Because we were analyzing a species-level phylogeny, we used a Yule tree prior, which assumes a constant speciation rate per lineage. We applied an uncorrelated relaxed molecular clock, with the substitution rate of the branch lengths being sampled from a prior normal distribution with a mean value of 0.02 and a standard deviation of 0.007 [94]. The search was conducted for a range of substitution rates that varied from 1% to 3% per million years. We ran four independent analyses for 20 × 10^6^generations. We checked for convergence and stationarity of the different analyses in Tracer 1.4 and combined the results in the BEAST module LogCombiner 1.4.4 (after removing the first 2 × 10^6 ^generations from each analysis as "burnin").

In analysis of the 16S rRNA, we modified the search strategy to overcome the slow rate of convergence and stationarity of the MCMC chains. We analyzed a matrix of 80 individuals and 512 bp. We first constructed a UPGMA tree with maximum likelihood distances (model selected in Mr.ModelTest v.2: TrN + I), which was specified as the starting tree in Beast. Previous studies have used values of 0.33% for the 16S rRNA or 0.7% more generally for a variety of different regions of the mitochondrial genome [e.g., [78,79]]. We specified the prior for the mean substitution rate as a normal distribution, with a mean of 0.008 and standard deviation of 0.003. This normal distribution thus covered the relevant range from 0.3% to 1% substitutions per site per My. We conducted two independent runs of 100 × 10^6 ^generations and combined the results with LogCombiner 1.4.4, after discarding the first 50 × 10^6 ^generations from the two runs as burnin. Results were checked using the program Tracer1.4.

### Morphometric analyses

We measured most (20 out of 22) of the morphometric traits described by Castellano and Giacoma [39]. As in that paper, we only included male toads: 17 from North Africa (from Morocco, Algeria, Tunisia, Egypt; collection ZFMK), 21 males of the new species from Sicily and the population means published by Castellano and Giacoma [39] on 118 male green toads from six populations from the Apennine Peninsula and NW-Italy [Vado L. (N = 18), Pellice (N = 45), Isolabella (N = 13), Maremma (N = 21), Calopezzati (N = 11), Leverano (N = 10)] and 56 male toads from the islands of Corsica [Cirindinu (N = 18)] and Sardinia [Barratz (N = 19), Portoscuso (N = 19)]. In the discriminant analyses, we initially included all 20 variables. However, as this number of variables probably represents an over-parameterization of a discriminant analysis with this number of specimens, we later reduced this number to five variables to test for its effect. For statistical analyses, the program SPSS 11.0 for Windows was used.

## Abbreviations

HPD = high probability density interval, mtDNA = mitochondrial DNA; MRCA = most recent common ancestor, My = Million years, Mya = million years ago; MVZ = Museum of Vertebrate Zoology, University of California, Berkeley, USA; ZFMK = Zoologisches Forschungsinstitut und Museum Alexander Koenig, Bonn, Germany; MZPA = Zoological Museum of Palermo University, Italy; NME = Naturkundemuseum Erfurt, Germany; ZMB – Naturkundemuseum of the Humboldt University, Berlin, Germany.

## Authors' contributions

MLoV, AS, MSt initiated collaboration; AS, MLoV, MSt, NMB performed field work; MSt, AS, SLoB sequenced mtDNA; MSt cloned and sequenced nuclear DNA, performed karyotyping, phylogenetic analyses; MSt, MLoV performed morphometric analyses and bioacoustics; SLoB, MA, MSt, and DB performed demographic analyses, DB performed divergence time estimates, MSt drafted the article and all author's provided text and improvements. All authors read and approved the final manuscript.

## Supplementary Material

Additional file 1 **Localities, specimen and voucher data and locality data**. Contains locality numbers as in Fig. 1, including geographic coordinates, GenBank accession numbers for the mitochondrial control region, the 16S rRNA, the RAG1-gene and the tropomyosine intron. Click here for file

Additional file 2 **Nomenclature**. This text (a) proposes vernacular names for *Bufo siculus *n.sp., (b) clarifies its etymology, (c) discusses synonymy and appropriateness of this new name according to International Code of Zoological Nomenclature, and (d) discusses the applicability of the name *Bufo balearicus *Boettger, 1880. The file includes references [96] to [104]. Click here for file

Additional file 3 **Green toads of other circum-Sicilian islands**. These are short biogeographic comments on green toads on Circum-Sicilian islands referencing the relevant literature. The file includes references [105] to [110]. Click here for file

Additional file 4 **Type description**. A verbal morphological description of the holotype of *Bufo siculus *n.sp. and a table containing morphometric data for the holotype and two adult paratypes. The file includes reference [111]. Click here for file

Additional file 5 **Permits**. Contains data for five collection permits for this study. Click here for file
