# Supplementary material for: Post-Messinian evolutionary relationships across the Sicilian channel: Mitochondrial and nuclear markers link a new green toad from Sicily to African relatives
Source: BMC Evol Biol. 2008 Feb 23;8:56. doi: 10.1186/1471-2148-8-56 (PMC2276203; doi:10.1186/1471-2148-8-56)
Supplement: Additional file 1 — Localities, specimen and voucher data and locality data. Contains locality numbers as in Fig. 1, including geographic coordinates, GenBank accession numbers for the mitochondrial control region, the 16S rRNA, the RAG1-gene and the tropomyosine intron. [file 1471-2148-8-56-S1.pdf]

	GenBank Acc.-No.	GenBank Acc.-No.	GenBank Acc.-No.	GenBank Acc.-No.					ADDITIONAL FILE 1, Page 1 of 2			
Locality number (as Fig. 1)	D-loop (or partial)	16S	Tropomyosine	RAG 1	Sample-ID	Voucher (if available)	N	Taxon	Locality	LAT	LONG	ELEVATION (if known)
1	DQ629730	-	-	-	371	-	1	B. boulengeri	Morocco, Ait Baha, E. Recuero leg.	30.130	-9.080	
2	DQ629718	-	-	-	112	MTD 45286	1	B. boulengeri	Morocco, High Atlas, D. Frynta leg.	32.427	-5.156	
3	DQ629704	-	-	-	179	CUP AMPH\MOR\01	1	B. boulengeri	Morocco, High Atlas, D. Frynta leg.	33.427	-5.150	1438
4	DQ629720	-	-	-	163	ZFMK 49652	1	B. boulengeri	Algeria, Ghardaia, W. Bischoff, U. Joger leg.	32.483	3.667	
5a	-	-	-	-	160	ZFMK 37856	1	B. balearicus	Spain, Balearic islands, Mallorca, Cape Andraixs, C.A. Raehmel leg. 1982	39.500	3.000	
5b	EU497593	-	-	-	81B		1	B. balearicus	Spain, Balearic islands, Mallorca, Artà, Charca temporal de Sa Vaca, J. Muntaner leg.	39.690	3.330	
5c	EU497594	-	-	-	82B		1	B. balearicus	Spain, Balearic islands, Mallorca, Fornalutx, Alberca de Bàlitx, J. Muntaner leg.	39.780	2.760	
5d	EU497595	-	-	-	83B		1	B. balearicus	Spain, Balearic islands, Mallorca, Sineu, Acequia carretera de Sineu a Petra, J. Muntaner leg.	39.640	3.030	
5e	EU497596<br>EU497597	-	-	-	84B, 85B		2	B. balearicus	Spain, Balearic islands, Mallorca, Llucmajor, Charca temporal de Son Cànaves, J. Muntaner leg.	39.480	2.880	
6	EU497577<br>EU497578<br>EU497579	EU497477<br>EU497478<br>EU497479	-	-	65B, 66B, 67B	-	3	B. balearicus	Spain, Balearic islands, Menorca, Torrellafuda, J. Pretus and A. Sicilia leg.	39.951	3.935	
7	EU497574<br>EU497575<br>EU497576	EU497474<br>EU497475<br>EU497476	-	-	62B, 63B, 64B	-	3	B. balearicus	Spain, Balearic islands, Menorca, Rafalet, J. Pretus leg.	39.841	4.298	
8	EU497568<br>EU497569<br>EU497570<br>EU497571<br>EU497572<br>EU497573	EU497468<br>EU497469<br>EU497470<br>EU497471<br>EU497472<br>EU497473	-	-	56B, 57B, 58B, 59B, 60B, 61B	-	6	B. balearicus	France, Corse, Cap Corse, Ersa, Barcaggio, M. Delaugerre leg.	43.050	9.408	
9	DQ629731	-	-	EU497604 (Bufo175)	175	ZSM 6/2004	1	B. balearicus	France, S-Corsica, near Bonifacio, F. Glaw, K. Schmidt, leg.	41.383	9.150	
10	EU497493<br>EU497494<br>EU497495<br>EU497496<br>EU497497<br>EU497498<br>EU497499<br>EU497500	-	EU497628, EU497629 (Bufo 395), EU497630 (Bufo 402)	EU497608 (Bufo 402)	395, 396, 397, 398, 399, 400, 402, 403	-	8	B. balearicus	Italy, NE-Sardinia, E of Monticanaglia, H. Veith leg.	41.061	9.531	
11	EU497563<br>EU497564<br>EU497565<br>EU497566<br>EU497567	EU497463<br>EU497464<br>EU497465<br>EU497466<br>EU497467	-	-	51B, 52B, 53B, 54B, 55B	-	5	B. balearicus	Italy, Sardinia, Sassari, Monte Nurra, G. Sotgiu leg.	40.723	8.342	
12	EU497562	EU497462	-	-	50B	-	1	B. balearicus	Italy, Sardinia, Oristano, Cabras, Stagno di Mistras, L. Bassu leg.	39.912	8.458	
13	EU497523<br>EU497524<br>EU497525	EU497423<br>EU497424<br>EU497425	-	-	11B, 12B, 13B	-	3	B. balearicus	Italy, Turine, Poirino, R. Sindaco leg.	44.922	7.822	
14	EU497518<br>EU497519<br>EU497520<br>EU497521<br>EU497522	EU497418<br>EU497419<br>EU497420<br>EU497421<br>EU497422	-	-	6B, 7B, 8B, 9B, 10B	-	5	B. balearicus	Italy, Pavia, San Martino Siccomario, F. Bernini leg.	45.155	9.142	
15	EU497526<br>EU497527	EU497426<br>EU497427	-	-	14B, 15B	-	2	B. balearicus	Italy, Pisa, Tenuta di San Rossore, A. Sicilia leg.	43.721	10.309	
16	EU497528<br>EU497529	EU497428<br>EU497429	-	-	16B, 17B	-	2	B. balearicus	Italy, Rome, Laurentina, A. Romano leg.	41.645	12.548	
17	EU497530<br>EU497532	EU497430<br>EU497432	-	-	18B, 20B	-	2	B. balearicus	Italy, Brindisi, Villa Castelli, T. Fattizzo leg.	40.578	17.461	
18	DQ629733	-	EU497622, EU497623 (Bufo 189)	EU497605 (Bufo 189)	189	NME 913/01	1	B. balearicus	Italy, W coast, Calabria, Paola, A. Nöllert leg.	39.350	16.033	
19	EU497533<br>EU497534<br>EU497535<br>EU497536<br>EU497537	EU497433<br>EU497434<br>EU497435<br>EU497436<br>EU497437	-	-	21B, 22B, 23B, 24B 25B	-	5	B. balearicus	Italy, Reggio Calabria, Condofuri, Amendolea, A. Sicilia leg.	37.983	15.894	
20	DQ629732	-	EU497620, EU497621 (Bufo 188)		188	NME 912/01	1	B. balearicus	Italy, Sicily, N of Francavilla di Sicilia, stream valley, T. Zavianni, A. Nöllert leg.	37.900	15.133	
21	DQ629726<br>DQ629727<br>DQ629728<br>DQ629729<br>EU497501<br>EU497502<br>EU497503<br>EU497504	-	EU497626 (Bufo 324), EU497627 (Bufo 325)	EU497609 (Bufo 325)	323, 324, 325, 326, 420, 421, 422, 423	ZMB 69556, MVZ 250741, MVZ 250742, MVZ 250743	8	B. siculus	Italy, Sicily, E of Lentini, near mouth of San Leonardo River, 500 m from coast to inland, M. Lo Valvo leg.	37.333	15.067	5
22	EU497543<br>EU497544<br>EU497545<br>EU497546<br>EU497547	EU497443<br>EU497444<br>EU497445<br>EU497446<br>EU497447	-	-	31B, 32B, 33B, 34B, 35B	-	5	B. siculus	Italy, Sicily, Agrigento, Macalube di Aragona, A. Sicilia leg.	37.377	13.596	

	GenBank Acc.-No.	GenBank Acc.-No.	GenBank Acc.-No.	GenBank Acc.-No.					ADDITIONAL FILE 1, Page 2 of 2			
23	EU497505<br>EU497538<br>EU497539<br>EU497540<br>EU497541<br>EU497542	EU497438<br>EU497439<br>EU497440<br>EU497441<br>EU497442	-	-	26B, 27B, 28B, 29B, 30B, 424	ZFMK 85896	6	B. siculus	Italy, Sicily, Palermo, Monte Pellegrino, A. Sicilia, M. Lo Valvo leg.	38.170	13.351	
23a	EU497598	-	-	-	86 B	MZPA A95	1	B.siculus	Italy, Palermo, La Fossa, A. Sicilia leg.	38.211	13.290	
24	EU497507<br>EU497508<br>EU497509<br>EU497510<br>EU497511<br>EU497548<br>EU497549<br>EU497550<br>EU497551<br>EU497552	EU497448<br>EU497449<br>EU497450<br>EU497451<br>EU497452	-	-	415, 416, 417, 418, 419, 36B, 37B, 38B, 39B, 40B	-	10	B. siculus	Italy, Ustica island, Gorgo di San Bartolicchio, A. Sicilia, M. Lo Valvo leg.	38.700	13.172	
25	EU497553<br>EU497554<br>EU497555<br>EU497556	EU497453<br>EU497454<br>EU497455<br>EU497456	-	-	41B, 42B, 43B, 44B	-	4	B. siculus	Italy, Favignana island, Cala Rossa, A. Sicilia leg.	37.921	12.360	
26	EU497512<br>EU497557<br>EU497558<br>EU497559<br>EU497560<br>EU497561	EU497457<br>EU497458<br>EU497459<br>EU497460<br>EU497461	EU497631, EU497632 (Bufo 425)	EU497612 (Bufo 425)	45B, 46B, 47B, 48B, 49B, 425	MVZ 250744	6	B. cf. boulengeri	Italy, Lampedusa island, Contrada Poggio Monaco, G. Nicolini and Mario Lo Valvo leg.	35.508	12.600	
27	EU497580<br>EU497581<br>EU497582	EU497480<br>EU497481<br>EU497482	-	-	68B, 69B, 70B	-	3	B. boulengeri	Tunisia, Cap Bon, Lebna, A. Sicilia leg.	36.728	10.931	
28	EU497583<br>EU497584	EU497483<br>EU497484	-	-	71B, 72B	-	2	B. boulengeri	Tunisia, El Kef, A. Sicilia leg.	36.166	8.700	
29	DQ629721	-	EU497616, EU497617 (Bufo 166)	EU497613 (Bufo 166)	166	MVZ 235680	1	B. boulengeri	Tunisia, Nefta oasis, Tawzar (=Tozeur) Governorate, T. Papenfuss leg.	33.917	8.133	45
30	EU497585<br>EU497586<br>EU497587	EU497485<br>EU497486<br>EU497487	-	-	73B, 74B, 75B	-	3	B. boulengeri	Tunisia, Kerkennah islands, Chergui, Remla, A. Sicilia leg.	34.704	11.200	
31	EU497588<br>EU497589	EU497488<br>EU497489	-	-	76B, 77B	-	2	B. boulengeri	Tunisia, Kerkennah islands, Chergui, El Kraten, A. Sicilia leg.	34.820	11.258	
32	DQ629719	-	-	-	165	ZFMK 14704	1	B. boulengeri	Tunesia, Djerba island, Kiehlmann leg. 1974	33.800	10.900	
33	EU497590<br>EU497591<br>EU497592	EU497490<br>EU497491<br>EU497492	-	-	78B, 79B, 80B	-	3	B. boulengeri	Tunisia, Tataouine, A. Sicilia leg.	32.903	10.416	
34	DQ629717	-	-	-	140	-	1	B. boulengeri	Libya, Al' Fjayj, Sabah Province, D. Frynta leg.	26.533	13.317	
35	DQ629705<br>DQ629706<br>DQ629707	-	-	-	139, 107, 114	-	2	B. boulengeri	Libya, Gabroon Lake, D. Frynta leg.	26.800	13.533	
36	DQ629710<br>DQ629711<br>DQ629712	-	EU497614, EU497615 (Bufo 109)	EU497607 (Bufo 109)	109, 131, 138	MTD 45036, 45281	3	B. boulengeri	Libya, Shahhat (Ancient Cyrene), Binghazi Province, D. Frynta leg.	32.817	21.867	
37	DQ629714<br>DQ629715	-	-	-	108, 110	MTD 45280, 45282	2	B. boulengeri	Egypt, Matrouh, via E. J. Bentley	30.000	28.000	
38	DQ629708<br>DQ629709	-	-	-	146, 147	ZFMK 77600, 77601	2	B. boulengeri	Egypt, Oasis Dakhla (Dakhilah, Al Wahat ad), N. Lutzmann leg.	25.553	28.948	
39	DQ629713	-	-	-	105	MTD 45277	1	B. boulengeri	Egypt, 70 km S Alexandria, via J. Bentley	31.000	30.000	
40	DQ629716	-	-	-	159	ZFMK 50909	1	B. boulengeri	Egypt, Alexandria, El Menoufia (via U. Sinsch), 1989	30.500	31.000	
41	DQ629687	-	-	-	21	-	1	B. viridis	Italy, Padua, University of Würzburg 1995 leg.	45.417	11.883	
42	EU497514<br>EU497516<br>EU497517	EU497415, EU497416,EU497417	-	-	3B, 4B, 5B	-	3	B. viridis	Italy, Trieste, San Dorligo della Valle, A. Sicilia leg.	45.621	13.869	
43	EU497515	EU497414	-	-	2B	-	1	old isolate	Croatia, Cres island, Belej, Museum of Natural History of Trieste coll.	44.766	14.429	
44	EU497513	EU497413	-	-	1B	-	1	old isolate	Croatia, Krk island, Stara Bas_a, Museum of Natural History of Trieste coll.	44.957	14.688	
45	DQ629686	-	EU497618, EU497619 (Bufo 168)	EU497603 (Bufo 168)	168	MVZ 164718	1	B. viridis	Austria, MVZ frozen tissue collection (FC 13312), 3.2 km E Podersdorf Burgenland, Austria; R. D. Sage leg.	47.850	16.833	
46	DQ629678	-	-	-	265	HNHM 2004.94.2	1	B. viridis	Hungary, Central Hungary, Orgovany, May 2004, L. Forro leg.	46.750	19.467	
47	DQ629722	-	-	-	149	ZFMK 62479	1	B. variabilis	Greece, Epirus, S Igoumenitsa, Patraia, W. Böhme leg. 1996	39.500	20.266	
48	DQ629630	-	-	-	99	-	1	B. variabilis	Greece, Peloponnes, J. Plötner leg.	37.516	22.367	
49	DQ629675	-	-	-	187	NME 901/01	1	B. viridis	Greece, Peloponnes, Kióna, E-Bank Stymphalian Lake, leg. A. Nöllert, 10 April 1996	37.850	22.450	
50	DQ629654	-	-	-	186	NME 900/01	1	B. viridis	Greece, Alepochori, motorway to Vactioni, leg. A. Nöllert, 2 April 1996	38.133	23.000	
51	DQ629655<br>DQ629656	-	-	-	133, 134	NME A 1037/03 (2nd + 3rd individual)	2	B. viridis	Greece, Crete, Omalos, U. Scheidt leg.	35.333	23.900	
52	DQ629657<br>DQ629658	-	-	-	135, 136	-	2	B. viridis	Greece, Crete, Aradena village, 19 April 2003, leg. U.Scheidt	35.200	24.083	
53	DQ629621<br>DQ629624	-	-	-	236, 237	MVZ 230206, 230207	2	B. variabilis	Turkey, Cicekli Köyü, 7 km E (by road) Ula Mugla Prov., T. Papenfuss leg.	37.066	28.500	
54	DQ629623	-	EU497624, EU497625 (Bufo 238)	EU497606 (Bufo 238)	238	MVZ 230208	1	B. variabilis	Turkey, Osman Gazi, Bursa, Bursa Prov., T. Papenfuss leg.	40.167	29.083	
55	EU497599<br>EU497600	-	-	-	87B, 88B	-	2	B. balearicus	Italy, Macerata, Morrovalle, M. Marconi leg.	43.280	13.586	
56	EU497601<br>EU497602	-	-	-	89B, 90B	-	2	B. balearicus	Italy, Macerata, Porto Recanati, N. Polini leg.	43.427	13.658	
						TOTAL:	148					
	-	-	-	EU497610	169	MVZ 186039	1	B. calamita	Spain, Cadiz Prov., Andalusia, 3.1 km S Benalup de Sidonia on road to Vejer de La Frontera, J.A. Visnaw leg.	36.333	5.817	
	-	-	-	EU497611	177905	MVZ 177905	1	Bufo bufo	Morocco, Marrakesh Prov., Oukaimeden, Stephen D. Busack, J. A. Visnaw	31.206	-7.864	2650
	-	-	EU497633	-	116	MTD 45287	1	B. raddei	China, Xinjiang, Kuku-Nor, J. Martens leg.	37.000	100.333	
	DQ629618	-	-	-	59	MTD 43944	1	B. surdus	Iran, Baluchestan, Deh Barez, D. Frynta leg.	27.450	57.317	350
