# Supplementary material for: Post-Messinian evolutionary relationships across the Sicilian channel: Mitochondrial and nuclear markers link a new green toad from Sicily to African relatives
Source: BMC Evol Biol. 2008 Feb 23;8:56. doi: 10.1186/1471-2148-8-56 (PMC2276203; doi:10.1186/1471-2148-8-56)
Supplement: Additional file 2 — Nomenclature. This text (a) proposes vernacular names for Bufo siculus n.sp., (b) clarifies its etymology, (c) discusses synonymy and appropriateness of this new name according to International Code of Zoological Nomenclature, and (d) discusses the applicability of the name Bufo balearicus Boettger, 1880. The file includes references [96] to [104]. [file 1471-2148-8-56-S2.pdf]

Additional file 2

Nomenclature

(a) *Proposed vernacular names for* Bufo siculus *n.sp.*

English: Sicilian green toad, Italian: Rospo smeraldino siciliano, German: Sizilianische Wechselkröte, French: crapaud vert de Sicile, Spanish: Sapo verde siciliano.

(b) *Etymology*

The Sicels (or Siculi) was one of the three tribes that originally inhabited Sicily and which gave Sicily the name it has held since antiquity. The Greek word *sikelos* ['native of Sicily'] became *sicolos* then *siculus* in Latin. We use *siculus* as a Latin adjective "siculus, -a, -um" = Sicilian, i.e. from Sicily.

(c) *On synonymy and appropriateness of the new name* Bufo siculus

Camerano's [96] taxonomic philosophy included the categories of species and subspecies (as exemplified by the name: "*Rana esculenta* Linn. subsp. *lessonae* var. nigro vittata", p. 190) and the hierarchical order and definitions of "variation", "variety", "subspecies" and "species" (op. cit., p.191). Camerano (p. 233 [96]) expressly gave his green toad variety names infrasubspecific rank (Art. 45.6.4. [97]), when he wrote: "In terms of the coloration, I believe one can establish the following main varieties" [emphasis added] of green toads in Italy, of which he observed some simultaneously in several regions of Italy (e.g., in Piedmont: var. *crucigera* and var. *F. lessona*). Therefore, these unequivocally intrasubspecific entities maintain this rank (and cannot be deemed subspecific, although published before 1961, Article 45.6.4. [97]). Camerano [96] not only quoted but also coined himself two such infrasubspecific entities, *concolor* for green toads from Piedmont, and *maculata*. On the latter he comments (transl. from Italian): "This variety is very frequent in all Italian localities; sometimes some of the spots on the back join and look like they give rise to longitudinal stripes (this fits some individuals from Modica - Sicily)". The stated occurrence all over Italy along with other such varieties reinforces its infrasubspecific status. In any case, according to ICZN (Art. 52 [97]) the name *maculata* is also invalid for this taxon since

it was a primary junior homonym of *Bufo maculatus* [98] at the time of Camerano's description [96] of *Bufo viridis* Var. *maculata*. Furthermore, to our knowledge, the type series is either lost or its location remains unclear (F. Andreone, curator of the Turin Museum, pers. comm.; cf. [99]). Therefore, we did not only coin the new name *Bufo siculus* but we also based it on a new type specimen (we could not acknowledge Camerano's work by designation of a Sicilian specimen from the syntype series of *Bufo viridis* Var. *maculata* as the holotype of *B. siculus*; as otherwise possible according to Paragraph 72.7 [97]).

(d) *On the applicability of the name* Bufo balearicus *Boettger, 1880*

The name coined by Boettger [45] for green toads from the Balearic Island of Mallorca seems currently the oldest available name clearly identifiable with this taxon, since sequences from Mallorca and Menorca appear nested in the clade that also contains the sequences from Sardinia, Corsica and most of the Apennine Peninsula. An old name (*B. viridis* var. *lineatus* [100]), coined for green toads from the Venice region (i.e., between our loc. 41 and 42), is a junior subjective synonym of *B. viridis* [101], as this phylogeographic group is present in that area. Although detailed descriptions of green toads from the range of what we consider *B. balearicus* date at least as far back as Cetti's [102] work, we did not find older scientific names for it, since neither Cetti [102], nor Gené [103] or de Betta [104] coined available scientific names for green toads.
