# Supplementary material for: Post-Messinian evolutionary relationships across the Sicilian channel: Mitochondrial and nuclear markers link a new green toad from Sicily to African relatives
Source: BMC Evol Biol. 2008 Feb 23;8:56. doi: 10.1186/1471-2148-8-56 (PMC2276203; doi:10.1186/1471-2148-8-56)
Supplement: Additional file 3 — Green toads of other circum-Sicilian islands. These are short biogeographic comments on green toads on Circum-Sicilian islands referencing the relevant literature. The file includes references [105] to [110]. [file 1471-2148-8-56-S3.pdf]

Additional file 3

Biogeographic comments on green toads on Circum-Sicilian islands

Green toads have been detected on the circum-Sicilian islands of Lipari, Salina, Ustica, Favignana, and Marettimo, and are known to have been introduced on Vulcano and Isola Grande dello Stagnone, but are probably not naturalized [41, 105, 106, 107]. A new report of a possible introduction on Pantellaria Island, of unknown origin, has been made [108]. No tissue sample for DNA analyses was collected, so the origin cannot be determined at this time. Gasc *et al.* [109] mapped green toads on Malta "after 1970". However, Savona Ventura (in litt.) reported that exclusively Pleistocene fossils have been found on Malta [110], perhaps from a colonization event out of Sicily during low sea levels, since the submarine plateau between Malta and Sicily is shallower than –95 m.
