# Supplementary material for: Post-Messinian evolutionary relationships across the Sicilian channel: Mitochondrial and nuclear markers link a new green toad from Sicily to African relatives
Source: BMC Evol Biol. 2008 Feb 23;8:56. doi: 10.1186/1471-2148-8-56 (PMC2276203; doi:10.1186/1471-2148-8-56)
Supplement: Additional file 4 — Type description. A verbal morphological description of the holotype of Bufo siculus n.sp. and a table containing morphometric data for the holotype and two adult paratypes. The file includes reference [111]. [file 1471-2148-8-56-S4.pdf]

Additional file 4

Morphological description of the holotype

P215, an adult female; for morphometric data see Table 5 (below), a large sized green toad; head distinctly shorter than wide, rather flat; snout slightly conical from lateral and dorsal view, not protruding; edges of interorbital space slightly wedge-shaped with the wider part orally, not much smaller than upper eyelid and little wider than internarial distance; nostril closer to tip of snout than to eye; tympanum large, distinct, vertical axis about more than half of eye diameter, anterior margin of tympanum closer to tip of snout than the posterior corner of eye, parotids almost adjoining the eyelid and adjacent to the upper margin of the tympanum; parotids wider than half their length, posterior part of the parotids slightly narrower and rounded, lower lateral edges of parotids reach the level of the upper third of the tympanum, small glands on the whole parotid surface; fingers not webbed, quite thick, their relative length (longest to shortest): 1, 3, 2, 4; tips of fingers rounded, not enlarged, subarticular tubercles prominent, single; two palmar tubercles, the inner about three times larger than the outer, numerous small tubercles covering the palms; hind limbs robust and relatively long, leg length more than 1.5 times tibia length, relative length of toes (longest to shortest): 4, 3, 2, 5, 1; tips of toes rounded, not enlarged, toes with dermal fringes, webbing only between the most proximal parts of toes, numerous rounded tubercles along the soles, subarticular tubercles single, inner metatarsal tubercle prominent, longish, about three times longer than wide; outer metatarsal tubercle only half as long as outer, longish; tarsal fold weak; dorsal and lateral skin with flat glandular warts of various size, lateral warts larger, snout and region between eyes smoother, dorsal surface of forelimbs and hindlimbs as well as ventral parts of forelimbs smooth; throat and belly without warts, but slightly warty; ventral parts of thighs with granular skin texture; coloration changed in preservation, in life darkly brownish olive spots of more than twice the eye diameter but with irregular shape (indentations) covered about three quarters of the dorsal skin, interspaces light (yellowish to greenish); ventral parts uniformly yellowish-whitish with numerous small grayish spots, especially between the hind limbs and towards the lateral margins.

Table 5: Morphometric data for the holotype and two adult paratypes.

	Sex	SVL	HL	PL	PW	HDT	VDT	ED	HW	IND	NED	TL	LL	LFT	LMT	IOW
P215	f	85.5	17.5	20.0	11.1	4.2	5.5	9.4	29.6	5.7	4.6	30.3	51.4	7.1	4.9	6.0
MZPA A95	f	69.7	16.4	15.6	8.7	4.1	5.0	7.5	26.4	5.5	3.5	27.0	45.7	6.6	4.1	5.2
ZFMK 8778	m	66.5	14.6	15.4	7.8	2.7	3.0	7.5	23.7	4.0	5.0	25.0	41.8	5.0	3.8	4.0

Abbreviations as in [111]: SVL: snout-urostyle length, HL: head length, PL: length of parotid gland, PW: width of parotid gland, HDT: horizontal diameter of tympanum, VDT: vertical diameter of tympanum, ED: horizontal diameter of eye, HW: head width, IND: internarial distance, NED: distance between nostril and anterior corner of eye, TL: length of tibia, LL: length of leg, LFT: length of first toe, LMT: length of inner metatarsal tubercle, IOW: interorbital width, f: female, m: male.
