# Supplementary material for: Post-Messinian evolutionary relationships across the Sicilian channel: Mitochondrial and nuclear markers link a new green toad from Sicily to African relatives
Source: BMC Evol Biol. 2008 Feb 23;8:56. doi: 10.1186/1471-2148-8-56 (PMC2276203; doi:10.1186/1471-2148-8-56)
Supplement: Additional file 5 — Permits. Contains data for five collection permits for this study. [file 1471-2148-8-56-S5.pdf]

Additional file 5

Permits

For this work, the following collection permits were kindly provided from the: Ministero dell'Ambiente e della Tutela del Territorio of Italy (DPN/2D/2005/12107) to M. Arculeo; Arrêté préfectoral (n°05-0336, 24 February 2005), Préfecture de Corse, France, to M. Delaugerre; Autoritzacio especial per a caça cientifica (Num. 11742/2006, 20 June 2006), Govern de les Illes Balears, Spain, to M. Stöck; the Ufficio legislativio e legale della Regione Siciliana Italy, (2005.49.3082, 15 November 2005) and Ente Gestore Rangers d'Italia (722/05, 22 August 2005) to M. Lo Valvo.
